# Rural houses infestation by *Triatoma infestans* in northwestern Argentina: Vector control in a high spatial heterogeneous infestation area

**DOI:** 10.1371/journal.pone.0201391

**Published:** 2018-08-02

**Authors:** María José Cavallo, Ivana Amelotti, Luciana Abrahan, Gerardo Cueto, David E. Gorla

**Affiliations:** 1 Entomología Médica, Centro Regional de Investigaciones Científicas y Transferencia La Rioja, UNLAR, SEGEMAR, UNCa, CONICET, Anillaco, La Rioja, Argentina; 2 Universidad Nacional de La Rioja, La Rioja, Argentina; 3 Instituto de Ecología, Genética y Evolución, Buenos Aires, Argentina; 4 Instituto de Altos Estudios Espaciales Mario Gulich, CONAE-Universidad Nacional de Córdoba, Córdoba, Argentina; Universite de Perpignan, FRANCE

## Abstract

*Triatoma infestans* (Hemiptera: Reduviidae) is a vector of the *Trypanosoma cruzi* parasite, causative agent of Chagas disease. During the last decade, vector control activities have been systematically carried out in northwestern Argentina, an endemic region for this disease. The general aim of this study to evaluate was spatio-temporal variation of infestation by *T*. *infestans* in rural communities of Los Llanos in La Rioja province. We estimated house infestation using two sampling methods: passive and active. Passive collection was conducted with community participation collecting triatomines. Six passive collections were carried out in 397 houses during the warm season between 2014 and 2017. Active collection of *T*. *infestans* was thoroughly performed by trained staff for 60 minutes and was carried out once in March 2016. The estimate of intradomestic infestation did not show significant differences between both collection methods (p = 0.39). However, passive collection method had lower sensitivity than active collection method for the estimation of peridomestic infestation and intradomestic colonization (PDI: p< 0.01; ID colonization: p< 0.01). The results obtained with passive collection methods showed that the infestation in the study area was spatially heterogeneous and temporally variable. Intradomiciliary infestation decreased over time (14.4% to 7.9%, p<0.05) although the effect of the chemical treatment application was not associated with the infestation level of *T*. *infestans* (p = 0.15) and the Departments had a different response each year (p<0.01). A high infestation cluster was located in the south of our study area during 2016–2017. The vector presence in the houses confirms the importance of to improve entomological surveillance programs. The search for triatomines carried out by the inhabitants might be a useful method to complement the activities of vector control programs in isolated and rural areas.

## Introduction

Triatomines are haematophagous insects that act as vectors of *Trypanosoma cruzi*, the causative agent of Chagas disease [1]. Although considerable progress has been made in the control of this disease, it is still major public health concern in Latin America [2]. In these areas, environmental and bio-socio-cultural factors favor the persistence of triatomine populations, allowing recolonization of domiciles even after vector control interventions with insecticide spraying [3, 4]. Among these factors, favorable climate conditions (warm temperature and low rainfall), high phenotypic plasticity and the capacity to adapt to different micro-geographic conditions favor the development of *Triatoma infestans* populations [5, 6]. In addition, these areas have a subsistence economy, with domestic animals (mainly goats and chickens) living in the immediate surroundings of the houses [7]. The fact that triatomine bugs frequently feed on these animals [8] increases the risk of *T*. *cruzi* vectorial transmission.

Insecticide based control of domestic vector populations still remains the core tool for prevention of new vectorial cases of Chagas disease [9, 10]. Trained teams of the “Programa Provincial Chagas de La Rioja (PPCHLR)” carry out periodic entomological evaluations within the houses and surrounding structures (corrals, coops and deposits), applying chemical interventions when needed. Within La Rioja, the high *T*. *infestans* infestation of rural houses in the region of Los Llanos is well known for the early work by Soler, during the 1950s [11]. The latest survey published, which collected data of over 5,045 houses in the area between 2004 and 2007, reported a 37% of house infestation (intradomestic and peridomestic infestation), after decades of non-systematic vector surveillance and control. In addition, a high spatial heterogeneity in *T*. *infestans* distribution has been reported specifically for this region, with highly infested localities concentrated in some areas [12]. In those regions it is necessary a fully operational long-term entomological surveillance system since, even after systematic control activities, residual house infestation is frequent [13].

Intra and peridomestic infestation by *T*. *infestans* is routinely estimated using an active fixed-time collection by trained staff of the vector control programs, sometimes using an irritant dislodging agent. Although adopted by all triatomine vector control programs in Latin America by the 1980s, it is well known that the active search has poor sensitivity when vector density is low, and its reliability depends upon the experience of collectors [14, 15]. Alternatively, it has been proposed that a more active involvement of the communities would be more suitable for rural and dispersed areas [16–18]. Passive collection implements the participation of the householders in the collection of triatomines and it has been used in a number of studies to estimate the levels of domestic infestation [14, 15, 17–19]. The advantage of this method is the relatively constant surveillance that the residents carried out for several days, increasing sensitivity [20], in particular, when the inhabitants are reluctant to allow the control team to search inside their houses.

Since the last report based on data from 2007 data on house infestation by *T*. *infestans* in rural communities of La Rioja [12], a number of changes occurred in the area, including the systematic operation of a provincial vector control program and the partial replacement of the traditional adobe-walled houses, which favors the presence of triatomines [21], by newer ones. For this reason, it is necessary to verify if the systematic interventions carried out during the last decade in Los Llanos region successfully controlled the vector.

In this context, the main objectives of our study were: 1) to compare active and passive collection methods to estimate the levels of *T*. *infestans* house infestation in rural communities of Los Llanos (La Rioja, Argentina), 2) to analyze the temporal variation (2014–2017) and spatial distribution of house infestation and 3) to identify high infestation areas within the study region. Altogether, this information would help the design novel and more cost-effective interventions for the sustainable control of Chagas disease and its vectors.

## Methods

### Study area

The study was carried out between 2014 and 2017 over 397 houses randomly selected in the provincial departments which showed the highest house infestation by *T*. *infestans* according to the latest study in the area [12]. The houses included in the study are located in the southern region of Los Llanos (La Rioja province, Argentina, (31° 21S, 66° 35’W)) and distributed along 72 rural localities in three departments (San Martín (SM), Rosario Vera Peñaloza (RVP) and Ángel Vicente Peñaloza (AVP)). Houses were extracted from the database built from 2005 by PPCHLR which georeferenced and identified all rural houses of La Rioja with an individual code engraved on a small metal plate attached to the front wall of each house. This georeferenced database allows an individual follow-up of the entomological condition and vector control intervention. Localities were defined as a group of houses separated at least two kilometers. The number of houses per locality varies in the area and 76.4% of them have six or fewer houses. This field study did not involve vertebrates and endangered or protected species. No specific permissions were required for our area or the activities developed in this work.

### House infestation by *Triatoma infestans*

House infestation was evaluated using passive and active collection methods. The collection method was defined as “passive” when the householders monitored house infestation. To this end, inhabitants of the selected houses received a detailed explanation of the study and were invited to participate. Inhabitants that accepted the invitation were trained in triatomine identification and careful collection to avoid the risk of accidental infection. Each participating family received two plastic bags labeled with the house identification code, one bag to collect triatomines inside the house and one to collect them in the peridomestic structures. Each passive collection lasted two weeks (between delivery and collection of the plastic bags). The passive collections were performed six times over three years, during spring (November 2014, 2015 and 2016) and summer (February 2015, 2016 and 2017). The number of houses assessed in each survey varied according to the year and season (from 256 to 341 houses) due to the fact that houses were closed during entomology evaluation or heavy rains that isolated the area.

The collection method was defined as “active” when a trained staff (a researcher and a PPCHLR technician) searched thoroughly for 15 minutes within the house and 15 minutes in peridomestic structures using an irritant dislodging agent (tetramethrin 0.2%, Espacial^®^). The active collection was conducted once in March 2016 in 78 houses evaluated with both methods (between February and March 2016). House selection was stratified for active sampling, considering the number of dwellings in each locality. Results obtained with both methods are considered independent because the people who performed the active collection did not know the previous infestation status of each house. However, this selection might be doubly biased since the same householders who accepted to collect triatomines in their home also enabled the active search. For this reason, the rates obtained over this sub-sample were only used to compare the collection methods. During the study period, the PPCHLR applied residual insecticide spraying in 121 of the 397 houses identified as infested during 2014 and 2015.

All triatomines were labeled in the field and then, in the laboratory, they were identified and quantified by species and developmental stage [22]. Rectal material of *T*. *infestans* specimens was analyzed under a microscope at 400x to detect the presence of *Trypanosoma cruzi*.

### Data analysis

A rural house was recorded as “infested” when at least one *T*. *infestans* individual was found in the house, without distinguishing whether the individual was an adult or a nymph. Additionally, a house was recorded as “colonized” when at least one 3^rd^, 4^th^ or 5^th^ *T*. *infestans* instar nymph was found in the house. According to the type of methodology carried out in this research and due to the fact that adult females frequently laid eggs in the bags after being collected, the presence of 1st and 2nd stage nymphs was not taken into account. We considered infestation or colonization in the intradomicile (IDI, IDC) or in the peridomicile (PDI, PDC) when it was recorded inside the house or in the nearby associated structures respectively (goat corrals, chicken coops, storerooms, etc). The percentages of infested houses and colonized houses were calculated over the total evaluated houses and expressed as “house infestation” and “house colonization” respectively. The statistical analysis was made with R statistical software (v. 3.4.3). The 95% confidence interval was fitted using the “binom.test” function to perform the exact test.

For the comparison of passive and active collection methods in order to estimate house infestation, we used Odds Ratio (OR) test and the Cohen’s kappa Index [23–25]. We used the active collection method as the reference method to compare with the performance of the passive collection method. Besides Cohen’s kappa, we calculated the sensitivity (proportion of infested houses identified by active collection method) and the specificity (percentage of uninfested houses identified by active collection method) [25]. To compare the infestation at departmental and locality levels and its variation over time, annual data obtained by passive collection were aggregated within each warm season (from November to February in two consecutive years): Year 1 (2014–2015), Year 2 (2015–2016) and Year 3 (2016–2017). To model changes in *T*. *infestans* infestation among year, season and department, a Generalized estimating Equation (GEE) was fitted using function geeglm function from package geepack [26] in the R statistical software (v. 3.4.3) [27]. Presence/absence of *T*. *infestans* in each house was included as the response variable (with a binomial error distribution and logit link function) while “year” (year1, year2 and year3), “season” (spring (November) and summer (February)) and “department” (SM, RVP and AVP) and their interactions were included as fixed factors. To control for possible differences generated by various intervals since the last chemical treatment (between 2 and 133 months), this was included as a covariable. This covariable was centered at the mean (average time of the last chemical treatment in the whole area) to improve the interpretation of model coefficients. Thus, the effects of the remaining variables were analyzed in groups of houses with the same previous treatment interval. A first order-autoregressive correlation structure was added to account for the non-independence among the repeated observations for the same house. Non-significant interactions were removed, one at a time from higher to lower levels, to reduce the number of parameters to be estimated. Mean comparisons were performed by Tukey's method using emmeans package [28].

Using the locality as the unit of analysis, a spatial scan statistic with a Poisson model was used to detect clusters (groups of localities geographically aggregated and with higher or lower infestation compared with the regional average). This analysis was performed using SaTScan v. 9.4.4.

## Results

During the study period, and combining the results of passive and active methods, we collected a total of 1395 *T*. *infestans* individuals. Five from the 912 individuals, which had enough blood to detect the presence of *T*. *cruzi* under microscope were infected with the parasite (4 adults and one 5th instar nymph) (S1 Table). House infestation (IDI + PDI) estimated by passive collection was 43.8% (174/397) and house colonization was 12.1% (48/397) at least once between November 2014 and February 2017 (Table 1). Most insects collected by passive collection method were adults (67.7%) and most insects collected by active method were nymphs (70.4%). Regarding the infested houses, up to 17 intradomestic specimens were found in a single house (being 2 specimens per house the most frequent number).

**Table 1:** Infestation and colonization by *Triatoma infestans* estimated by passive collection in Los Llanos, La Rioja.

Date	Evaluated houses	House infestation % (CI95)	IDI% (CI95)	PDI% (CI95)	IC % (CI95)	SD
Nov2014	341	21.4 (17.2–26.1)	14.4 (10.8–18.5)	3.5 (1.8–6.1)	3.2 (1.6–5.7)	5.6
Feb2015	256	13.7 (9.7–18.5)	6.6 (3.9–10.4)	3.9 (1.9–7.1)	0.6 (0.1–2.1)	5.1
Nov2015	322	14.3 (10.7–18.6)	8.4 (5.6–12.0)	2.2 (0.9–4.4)	1.2 (0.3–3.2)	8.4
Feb2016	320	10.9 (7.7–14.9)	3.4 (1.7–6.2)	4.1 (2.2–6.8)	1.5 (0.5–3.4)	6.3
Nov2016	291	13.7 (10.0–18.2)	6.5 (4.0–10.0)	2.1 (0.8–4.4)	0.3 (7*e*-3–1.6)	4.8
Feb2017	289	11.1 (7.7–15.3)	7.9 (5.1–11.7)	3.1 (1.4–5.8)	2.6 (1.2–5.0)	0

Data correspond to six sampling periods from 2014 to 2017. IDI, percentage of intradomestic infestation; PDI, percentage of peridomestic infestation; IC, percentage of intradomestic colonization. CI, 95% confidence intervals are given in brackets. Evaluated houses are the number of houses that returned collection bags; House infestation is the percentage of houses that returned collection bags with at least one *T*. *infestans*; Intradomestic colonization is the percentage of houses that returned collection bags with with 3^rd^, 4^th^ or 5^th^ *T*. *infestans* instar nymphs. SD: Triatomines collection place (intra o peridomestic) is unknown.

House infestation (IDI + PDI) estimated by active collection was higher than the estimated by passive collection (Table 2). For active collection methods, the PDI was higher than the IDI (64.1% (50/78 houses) vs. 20.5% (16/78), p< 0.01). House colonization was almost seven times higher when it was estimated by active collection compared to passive collection (60.3 vs. 8.9% respectively). When householders did not indicate the collection place of the triatomines (intra or peridomestic) the data were not considered in the analyses. When discriminating the number of *T*. *infestans* collected by active method per ID and PD structures, the highest abundances were showed in PD structures that included chicken coops, goat corrals and storerooms. Storerooms, dog houses and hen nests showed the highest average abundances (Table 3).

**Table 2:** Comparative statistics of estimated parameters of intra and peridomestic infestation and colonization between active (March 2016) and passive (February 2016) collection methods (n = 78 houses).

	IDI%	PDI%	IDC%
Active collection method (CI)	20.5 (11.5–29.5)	64.1 (53.5–74.7)	60.3 (49.4–71.1)
Passive collection method (CI)	14.1 (6.4–21.8)	16.7 (8.4–25.0)	8.9 (2.6–15.3)
Kappa (Standard error)	42.2 (1.6)	20.1 (1.1)	7.8 (1.1)
Sensitivity	43.7	26	12.8
Specificity	93.5	100	96.8

IDI, Intradomestic infestation; PDI, Peridomestic infestation; IDC, Intradomestic colonization; CI, 95% Confidence Interval. Active search is used as the reference for sensitivity and specificity.

**Table 3:** Number of *Triatoma infestans* collected in intradomestic and peridomestic structures using active collection methods (March 2016).

Structure type	No. of evaluated structures	Abundance of *T*. *infestans*	Mean abundance per habitat (minimum and maximum values)
Chicken coops	26	220	8.46 (2–39)
Goat corrals	30	186	6.2 (1–94)
Storerooms	12	133	11.08 (4–28)
Hen nests	4	58	14.5 (8–15)
Dog houses	3	38	12.67 (12–14)
Houses	16	22	1.38 (1–7)
Pigeon houses	1	5	5

Abundance is the total number of individuals collected in each structure type from of 78 evaluated houses.

The agreement between the estimates using passive and active collection methods varied with the type of parameter considered. The concordance using the Kappa index of IDI (when given positive by the two methods) was 42.2 (Table 2). Intradomestic infestation showed no significant differences when it was estimated by active or passive collection methods (OR = 0.63, 95% IC = 0.27–1.48, χ2 = 0.7, df = 1, p = 0.4). However, peridomestic infestation and intradomestic colonization showed a significant underestimation by the passive method (PDI: OR = 0.11, 95% IC = 0.05–0.24, χ2 = 34.5, df = 1, p< 0.01; ID colonization: OR = 0.07, 95% IC = 0.02–0.16, χ2 = 43.1, df = 1, p< 0.01). The sensitivity was higher for the IDI than for PDI and more than 93% of the houses reported uninfested by householders were corroborated for active collection method, showing the passive collection method a high specificity (Table 2, S2 Table).

**Fig 1:**
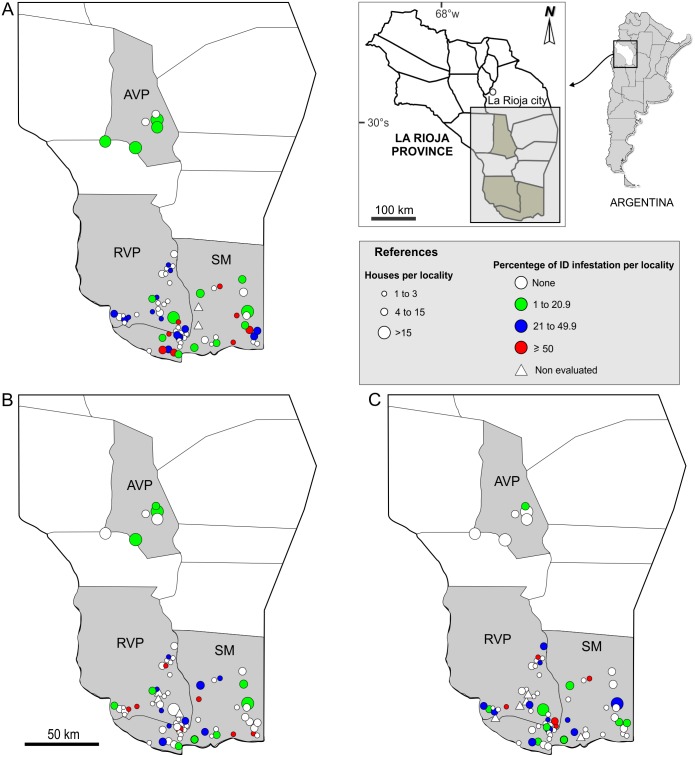
Variation over three years of the intradomestic infestation by *Triatoma infestans* estimated by passive collection in three departments of Los Llanos, La Rioja. (A) Year 1 (data November 2014 and February 2015). B) Year 2 (data November 2015 and February 2016). (C) Year 3 (data November 2016 and February 2017). Grey shadows indicate the evaluated departments: AVP, Ángel Vicente Peñaloza Department; RVP, Rosario Vera Peñaloza Department; SM, San Martín Department.

**Fig 2:**
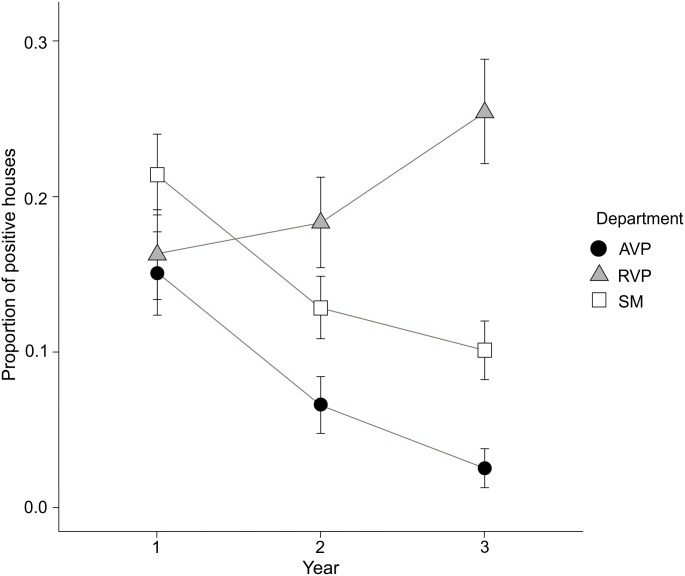
Temporal variation of infestation by *Triatoma infestans* estimated by passive collection in each department. AVP, Ángel Vicente Peñaloza Department; RVP, Rosario Vera Peñaloza Department; SM, San Martín Department. Lines over the bars are standard deviations. Year 1 (data November 2014 and February 2015). Year 2 (data November 2015 and February 2016). Year 3 (data November 2016 and February 2017).

At the locality level, 94.4% of the localities showed house infestation at least once within the 3-year period. Among the 72 studied localities, 23 showed an IDI higher than 50%, with many of them remaining positive over the whole study period (Fig 1). Infestation by *T*. *infestans* during the study period was associated with the "month", "department", "year" and "interaction between year and department". A higher infestation was found at the beginning of the warm season (November) (p<0.01) (Table 4). At the department level, the lowest average infestation (PD+ID) was in AVP, with a significant tendency to decrease during the three years of study, starting with 15% of houses infested. However, the department had a different response in each year, RVP and SM showed an average house infestation of 18.5% during the studied period, although it showed a significant decrease in SM and an increase in the third year in RVP (Fig 2). The time since the last chemical treatment for each house it was not associated with *T*. *infestans* infestation (p = 0.15) (Table 4, S3 Table).

**Table 4:** Wald chi-square tests for sequentially adding terms to the Generalized estimating Equation (GEE) for infestation by *Triatoma infestans*.

	House infestation		
Factor	Degree of freedom	χ2	p(> | Chi (χ2)|)
Year	2	7.74	0.0209*
Department	2	25.07	3.6e-06*
Season	1	9.05	0.0026*
Trult.c	1	2.07	0.1507
Year:Department	4	27.18	1.8e-05*

*, Overall significance level p˂0.05; Trult.c, time since the last chemical treatment for each house (centered at the mean).

**Fig 3:**
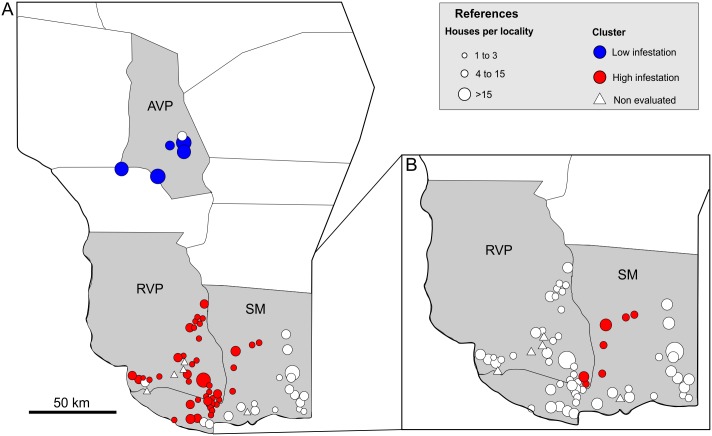
Spatial distribution of high and low intradomestic infestation clusters of rural localities in the study area. High and low intradomestic infestation aggregations (red and blue, respectively). AVP, Ángel Vicente Peñaloza Department; RVP, Rosario Vera Peñaloza Department; SM, San Martín Department. A. Clusters within AVP, SM and RVP localities. B. Clusters within SM and RVP localities.

The results of the spatial scan statistic discriminated by year showed that the IDI at the locality level in the study area was spatially heterogeneous and temporally variable. Years 1 and 2 showed no evidence of spatial aggregation, but two geographic clusters were detected during Year 3, when the total average ID infestation in the whole study area was 10.2%. One cluster included 113 houses in five localities within the AVP department (radius: 24.9 km centered at 30.65° S, 66.67° W) showed no house infestation (Relative risk = 0; p< 0.001) (Fig 3A). The second cluster included 161 houses in 43 localities. It showed an intradomestic infestation of 19.9% (Relative risk = 5.7; p< 0.001) and had a radius of 41.4 km, centered at 31.58° S, 66.51° W. This cluster consisted of localities from SM and RVP departments (Fig 3A). When analyzing the spatial heterogeneity using only the localities included in RVP and SM, it was found a cluster of high infestation with an IDI of 48% (Relative risk = 4.04; p = 0.035) which included 25 houses in seven localities within a radius of 19.6 km, centered at 31.66° S, 66.20° W (Fig 3B).

## Discussion

For the first time, house infestation by *T*. *infestans* in northwestern Argentina (Los Llanos, La Rioja) was estimated with rural community participation in a spatially heterogeneous infestation area. Additionally, the passive collection performed by householders was useful in providing a thorough record of other triatomine native species (e.g *T*. *garciabesi*, *T*. *guasayana*, *T*. *platensis*), which in general cannot be obtained with the active collection method [29].

Our study shows that the estimation of intradomestic infestation by passive collection was similar to active search. However, these results could probably be doubly biased since the same householders who accepted to collect the triatomines in their home also enabled the active search [30]. The main difference in the estimation of house infestation between both collection methods was the low sensitivity of passive collection to detect intradomestic colonization and peridomestic infestation. In intradomiciles, colonization estimates by passive collection showed that most of the collected insects were adults, easier to visualize for the householders. In contrast, the trained team who carried out the active collection is capable to visualize all the development stages. In peridomiciles, the estimation of infestation was lower by passive collection because householders probably spend more time in intradomiciles doing their day-by-day activities than in the peridomestic structures. Nevertheless, in active collection method, the search time in each place is standardized. Although the capture effort between collection methods used in this work was different, our result contrasts with previous reports showing that passive collection of *T*. *infestans* was more sensitive than the active search [15, 18, 20]. Moreover, other studies performed using active collection reported that corrals were the most important peridomiciles for *T*. *infestans* development [31, 32], while in our study, hen nests, dog houses and storerooms showed the highest average abundance of triatomines.

At season level, in November (Spring), house infestation by *T*. *infestans* was higher than in February (Summer). These results differ from empiric models which predict that the spread *T*. *infestans* is likely to take place at the end of summer [33]. In the present work, there was a decrease in house infestation during the study period, except in RVP department where isolated rural localities exist (lower number of houses per locality). Previous studies in the same area, showed that house infestation was inversely correlated with the number of houses in each locality [12]. Our data show that the chemical treatments carried out by the PPCHLR in this area during the study period have no effect on house infestation.

House infestation considered at departmental and locality levels, was spatially heterogeneous and temporally variable. Spatial aggregation of house infestation was not detected during the first two years of the study, but it did show up during the third year of sampling (2016–2017), when a cluster of high house infestation was detected in the southern extreme of the sampled area. The detected cluster is centered 25 km from the cluster of high domestic infestation previously reported in the same area [12], suggesting the presence of an unresolved focus of house infestation. The temporal variability of the vector presence reinforces the importance of continuity of the surveillance and control programs.

A decade-long follow up of house infestation based on data produced by the PPCHLR showed that 53.7% of the houses in the same region were infested during 2004–2005 (35.6% IDI and 43.8% PDI) [32]. A decade later (2014–2015), it was reported that house infestation was 10.3% for the IDI and 33.1% for the PDI (PPCHLR, unpublished data).

During this decade, the evaluated house coverage has been highly heterogeneous (PPCHLR, unpublished data). Not all localities were visited each year, and the average visit frequency to each house was once every three years. The low frequency of house evaluation added to the low effectiveness of insecticide spraying in peridomestic structures [34], undermined a sustained success of the PPCHLR. Although the activities of the decade-long vector control program carried out in Los Llanos decreased house infestation by *T*. *infestans* in an endemic area, this infestation was not eliminated, even when old and precarious rural houses were partially replaced. In addition, the collection of *T*. *infestans* infected with *T*. *cruzi* using both methods inside the houses reflects the risk of vector transmission of Chagas disease. The data presented in this work are lower compared to the situation in other areas [35–37], however, it is necessary to increase efforts to eliminate infected insects from homes.

Under this scenario, novel approaches for vector control programs and more cost-effective interventions to improve the sustainable control is needed. For this reason, the present study showed the advantage-disadvantage of the local community involvement. As repeatedly shown in many studies [15, 18, 19, 38–43], entomological surveillance carried out with the community participation in intradomiciles may promote earlier detection of vector infestation.

The estimation of intradomestic infestation by passive collection was as sensitive as by active search in intradomiciles and this method could be an important benefit for the country’s health system due to its cost-effectiveness. Future studies are necessary to analyze the difference in costs between active and passive collection methods. Moreover, in this study, passive search was useful to identify house infestation at a department and locality levels as well as to follow its variation over time. An important limitation to the success of this approach is that a commitment of health system is necessary to give a timely response and intervention when householders report that a house is infested in ID. The health system should guarantee the chemical treatment of house infestation by *T*. *infestans*. Otherwise, it will not be possible to maintain entomological surveillance by the householders over a long period of time.

As a conclusion, we propose that passive collection in conjunction with the traditional active search might significantly increase triatomine detection in the intradomestic environment, thus increasing the efficacy of *T*. *infestans* control.

## Supporting information

S1 Table *Triatoma infestans* bugs infected with *Trypanozoma cruzi* collected by two different collection methods in Los Llanos (La Rioja, Argentina) 2014–2017. (DOC)

S2 Table Contingency table of passive and active collection method on the houses evaluated by both collection methods (n = 78). (DOC)

S3 Table Parameter estimates of generalized estimation (GEE) equation models for *T*. *infestans* infestation. (DOC)
