# Supplementary material for: Rural houses infestation by Triatoma infestans in northwestern Argentina: Vector control in a high spatial heterogeneous infestation area
Source: PLoS One. 2018 Aug 2;13(8):e0201391. doi: 10.1371/journal.pone.0201391 (PMC6072006; doi:10.1371/journal.pone.0201391)
Supplement: S1 Table — (DOC) [file pone.0201391.s001.doc]

**S1 Table. *Triatoma infestans* bugs infected with *Trypanozoma cruzi* collected by two different collection methods in Los Llanos (La Rioja, Argentina) 2014–2017.**

| **Date** | **Department** | **Locality** | **Collection**  **method** | **Amount/n** | **Ecotope** | **Latitude** | **Longitude** |
| --- | --- | --- | --- | --- | --- | --- | --- |
| November 2014 | San Martín | San Solano | Passive | 2 adults / 8 | Intradomicile | -31.47833 | -65.9202 |
| February 2016 | Rosario Vera Peñaloza | Totoral | Passive | 1 adult / 4 | Intradomicile | -31.70972 | -66.40377 |
| March 2016 | San Martín | Balde de la Viuda | Active | 1 5th instar nymph / 31 | Intradomicile | -31.84075 | -66.47343 |
| March 2016 | San Martín | La Reserva | Active | 1 adult / 15 | Peridomicile | -31.85848 | -66.09264 |

RVP**,** Department of Rosario Vera Peñaloza; SM, Department of San Martín; n: total of *Triatoma infestans* (nymphs + adults) by house and collection method.
