# Supplementary material for: Rural houses infestation by Triatoma infestans in northwestern Argentina: Vector control in a high spatial heterogeneous infestation area
Source: PLoS One. 2018 Aug 2;13(8):e0201391. doi: 10.1371/journal.pone.0201391 (PMC6072006; doi:10.1371/journal.pone.0201391)
Supplement: S2 Table — (DOC) [file pone.0201391.s002.doc]

**S2 Table. Contingency table between passive (February 2016) and active (March 2016).**

**S2A Table. Intradomestic infestation**

|  |  | **Active collection method** | | |
| --- | --- | --- | --- | --- |
| **Passive collection method** |  | **+** | **-** | **Passive estimation (%)** |
|  | **+** | 7 | 4 | 14.1 |
|  | - | 9 | 58 | 85.9 |
|  | **Active estimation (%)** | 20.5 | 79.5 |  |

**S2B Table. Peridomestic infestation**

|  |  | **Active collection method** | | |
| --- | --- | --- | --- | --- |
| **Passive collection method** |  | **+** | **-** | **Passive estimation (%)** |
|  | **+** | 13 | 0 | 16.7 |
|  | **-** | 37 | 28 | 83.3 |
|  | **Active estimation (%)** | 64.1 | 35.9 |  |

**S2C Table. Intradomestic colonization**

|  |  | **Active collection method** | | |
| --- | --- | --- | --- | --- |
| **Passive collection method** |  | **+** | **-** | **Passive estimation (%)** |
|  | **+** | 6 | 1 | 9.0 |
|  | **-** | 41 | 30 | 91.0 |
|  | **Active estimation (%)** | 60.3 | 39.7 |  |

n=78.
