# Supplementary material for: Rural houses infestation by Triatoma infestans in northwestern Argentina: Vector control in a high spatial heterogeneous infestation area
Source: PLoS One. 2018 Aug 2;13(8):e0201391. doi: 10.1371/journal.pone.0201391 (PMC6072006; doi:10.1371/journal.pone.0201391)
Supplement: S3 Table — (DOC) [file pone.0201391.s003.doc]

**S3 Table. Generalized estimating Equation (GEE).**

| **Factor** | **Coefficient estimate (Standard Error)** | **p** |
| --- | --- | --- |
| (Intercept) | -1.878 (0.258) | 3.440e-13*** |
| Year year2 | -0.894 (0.386) | 0.020* |
| Year year3 | -1.901 (0.549) | 0.001*** |
| Depto RVP | -0.086 (0.341) | 0.800 |
| Depto SM | 0.300 (0.297) | 0.311 |
| Season Spring | 0.408 (0.134) | 0.002** |
| Trult.c | -0.003 (0.002) | 0.053 |
| Year year2 : Depto RVP | -0.003 (0.002) | 0.026* |
| Year year3 : Depto RVP | 2.539 (0.613) | 3.430e-05*** |
| Year year2 : Depto SM | 0.330 (0.455) | 0.468 |
| Year year3 : Depto SM | 1.005 (0.608) | 0.098 |

QIC: 1439. Signif. Codes: 0 ‘***’ 0.001 ‘**’ 0.01 ‘*’ 0.05. Department; RVP, Rosario Vera Peñaloza Department; SM, San Martín Department; Trult.c, time since the last chemical treatment for each house (centered at the mean)**.**
